# Phenotypic analysis of extracellular vesicles: a review on the applications of fluorescence

**DOI:** 10.1080/20013078.2019.1710020

**Published:** 2020-01-07

**Authors:** Maria S. Panagopoulou, Alastair W. Wark, David J S Birch, Christopher D. Gregory

**Affiliations:** a University of Edinburgh Centre for Inflammation Research, The Queen’s Medical Research Institute, Edinburgh, UK; b Centre for Molecular Nanometrology, Technology and Innovation Centre, Department of Pure and Applied Chemistry, University of Strathclyde, Glasgow, UK; c Photophysics Group, Department of Physics, SUPA, University of Strathclyde, Glasgow, UK

**Keywords:** Exosomes, cancer, fluorescent dye, quantum dot, nanobody, fluorescent protein, microscopy, fluorescence lifetime, nanoparticle tracking analysis, super resolution microscopy, FLIM, fluorescence depolarization, FRET, qPCR, microfluidics, microarrays

## Abstract

Extracellular vesicles (EVs) have numerous potential applications in the field of healthcare and diagnostics, and research into their biological functions is rapidly increasing. Mainly because of their small size and heterogeneity, there are significant challenges associated with their analysis and despite overt evidence of the potential of EVs in clinical diagnostic practice, guidelines for analytical procedures have not yet been properly established. Here, we present an overview of the main methods for studying the properties of EVs based on the principles of fluorescence. Setting aside the isolation, purification and physicochemical characterization strategies which answer questions about the size, surface charge and stability of EVs (reviewed elsewhere), we focus on available optical tools that enable the direct analysis of phenotype and mechanisms of interaction with tissues. In brief, the topics on which we elaborate range from the most popular approaches such as nanoparticle tracking analysis and flow cytometry, to less commonly used techniques such as fluorescence depolarization and microarrays as well as emerging areas such as fast fluorescence lifetime imaging microscopy (FLIM). We highlight that understanding the strengths and limitations of each method is essential for choosing the most appropriate combination of analytical tools. Finally, future directions of this rapidly developing area of medical diagnostics are discussed.

## Introduction

### Extracellular vesicles

The first observation of mammalian vesicle-like structures was in 1967 by P. Wolf in platelet-free plasma after ultracentrifugation [1]. After years of extensive research, it is now well established that vesicles are released by cells to the extracellular milieu, serving various purposes such as cell-to-cell communication, exchange of genetic information and protection – the elimination of dangerous substances and unwanted metabolites such as chemotherapeutics or oxidized lipids from cells [2].

Mammalian EVs are secreted membrane-delimited structures that contain material from the cytosol, subcellular organelles and/or nuclear components, encapsulated in a lipid bilayer. Although it is unknown whether all cell types can secrete EVs [3], diverse cell lineages have proven capacity to do so. The process of vesicle secretion seems to be conserved throughout the evolution timeline [4], providing evidence that EVs have a specific role in the function of cells and organisms. The size (diameter) of EVs is not uniform, as they can range from around 30 nm up to a few micrometres. They have been detected in most body fluids such as blood, urine, amniotic fluid, cerebrospinal fluid, saliva, breastmilk and even nasal secretions. It is now believed that EVs provide important intercellular communication signals both in physiological and pathological situations such as cancer and neurodegenerative diseases [5].

From the biochemical perspective, EVs and their cargoes comprise several components, ranging from membrane lipids and cytosolic proteins to nucleic acids. More specifically, several techniques including Western blotting and immune electron microscopy have revealed the presence of proteins on the surface as well as the interior of EVs, some of which are ubiquitous (e.g. actin, kinases, heat-shock proteins) while others vary significantly depending on the cell type from which they originate, mainly lineage delineating proteins and disease-specific markers. A few examples of tissue-specific proteins are, among others: major histocompatibility complex MHC class II and CD86 for EVs derived from antigen-presenting cells in vitro [6], carcinoembryonic antigen (CEA) for EVs derived from colon carcinoma cell culture [7], human epidermal growth factor receptor 2 (HER2) from breast carcinoma cell lines [8] and prostate cancer antigen-3 (PCA-3) as well as TMPRSS2:ERG from EVs isolated from prostate cancer patients’ urinary samples [9]. Another basic structural component in all vesicles is the lipids, with some of the most common being phosphatidylcholine (PC), sphingomyelin, cholesterol and saturated fatty acids. Interestingly, nucleic acids have also been found in extracellular vesicles; most commonly small RNA molecules such as mRNA, different sizes of miRNA and low levels of ribosomal RNA. DNA has also been found, not only in larger vesicles which derive from apoptotic cells (apoptotic bodies) but in smaller EVs as well [10,11]. Thus, genetic material exchange implies a major role in cell-to-cell communication and disease regulation. This basic biochemical composition can vary significantly under particular micro-environmental conditions such as tumours and inflammation [4].

Because of their cargoes, EVs can be considered as vehicles for disease biomarkers which have advantages over routine blood biomarkers in common use [12]. Additional clinical applications of EVs are also currently under investigation. For example, EVs have shown intrinsic therapeutic activity when isolated from one specific tissue and administered to another, as, for example, shown in the case of mesenchymal stem cell-derived EVs which can have cardio-protective properties [13]. Furthermore, vesicles isolated from lymphoma models had pro-inflammatory properties, triggering systemic immune responses, indicating a potential use as vaccines [14]. Therefore, effective analysis of EV phenotypes is essential for understanding the properties of EVs and for shaping their potential clinical use.

### Challenges in EV analysis

Some of the most common difficulties in analysing EVs are:

i. small size and biochemical complexity;
ii. pre-analytical methodologies for isolation and purification, as the accuracy of the measurements often depends on the purity of the sample, especially for techniques with high sensitivity;

Typical isolation methods include gel permeation chromatography, ultracentrifugation, immunoaffinity capture or polymer-facilitated precipitation, among others [12]. A variety of properties are used for EV isolation, such as size, density, surface charge, hydrophilic interactions with solvents and affinity for biological targets [15]. In a recent approach, both capture and analysis of EVs on the same device are based on microfluidics and arrays (see later). Each technique has limitations and combinations of methods are therefore often used. However, in complex biological samples such as blood plasma, achieving highly pure EV preparations is especially challenging because of contamination of the EVs with particulates such as large protein aggregates (mostly lipoproteins) and chylomicrons, micelles of lipids and proteins that cannot be reliably removed by common purification methods such as centrifugation or size exclusion chromatography [16,17]. Moreover, the protocols for isolation and enrichment of EV samples have not been standardized as yet, posing limitation on the wide use of EVs in clinical settings [18].

More challenges in studying EV include:

iii. heterogeneity of EV populations;
iv. sub-populations of different phenotypes;
v. unknown concentrations;
vi. correlating specific EVs with specific origins;

A number of the above hurdles can be addressed by optical methods, on which this review focuses. Optical methods use light in order to read the information encoded in the sample. The fundamental advantage of optics-based techniques over calorimetric or chemical readout methods is that, because light appears in a spectrum of wavelengths, multiplexed analysis is permitted, which is essential for clinical samples where assessment of multiple molecules may be required. Moreover, with the selection of the appropriate fluorophore and target molecule, the different populations and origins of EVs can be studied, while it is also possible to precisely detect and localize biomolecules using super-resolution microscopy, excluding particles which cause impurities. Additionally, we acknowledge that non-optical technologies (of which there are many) can answer critical questions and common approaches incorporate both; non-conventional tools and future approaches will also be discussed briefly later (see Emerging fluorescence methods).

### Why optical methods?

**Figure 1.**
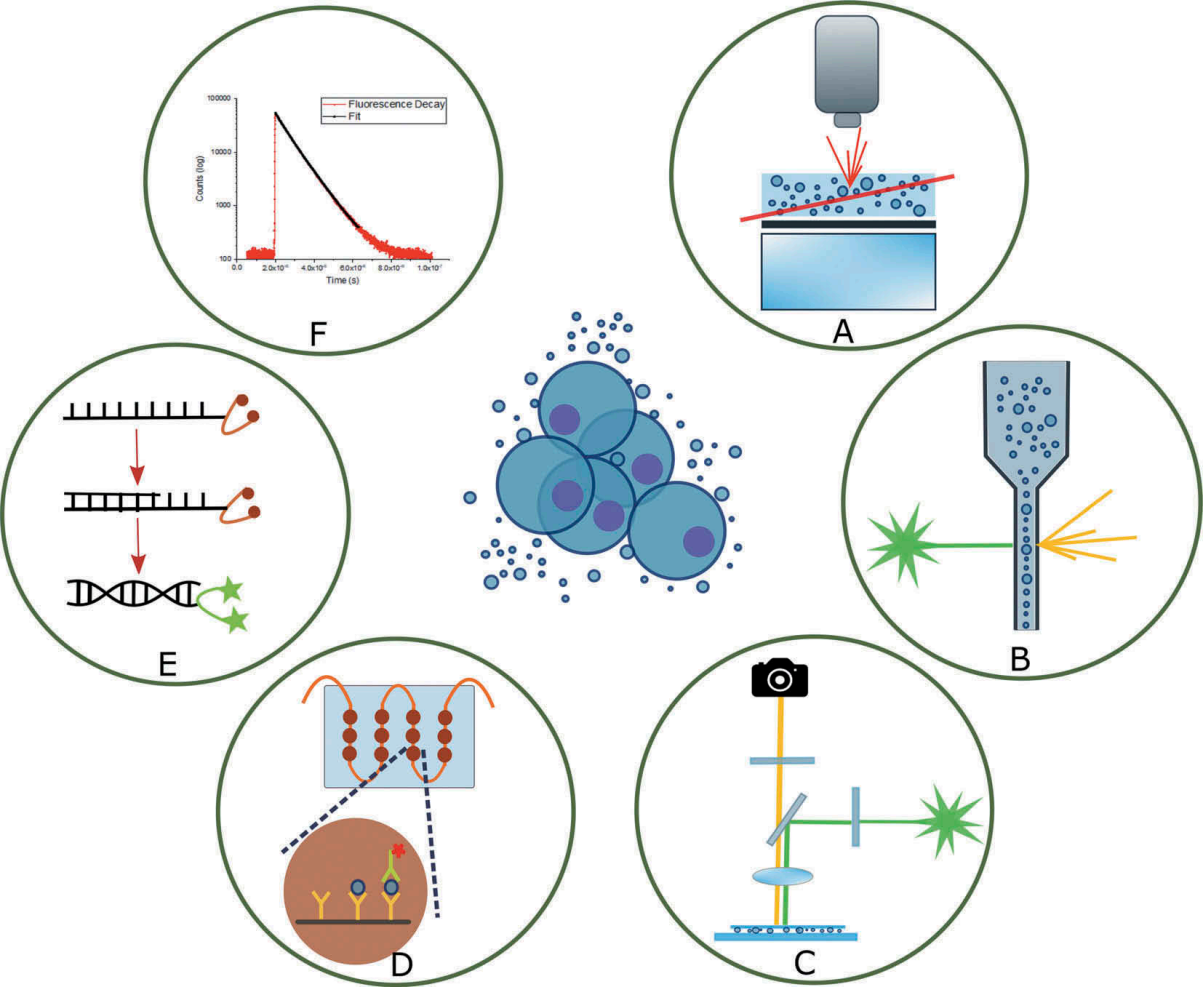
Simplified schematic overview of the main optical techniques used for EV analysis. Centre image: EVs secreted from cells. Techniques where EVs are analysed in suspension include A: nanoparticle tracking analysis, B: flow cytometry, C: fluorescence spectroscopy (lifetime, polarization). Methods where EVs are immobilized on a surface include D: fluorescence microscopy for isolated EVs (for live imaging, EVs can be in suspension) and E: microarrays and microfluidics. Also, F: quantitative PCR, in which the EVs are lysed.

The aim of this review is to summarize the optical tools researchers have used in analysing the phenotype and functions of EVs, emphasizing the seemingly endless potential of fluorescence-based approaches. In general, optical methods provide unique opportunities for real-time, multiplexed and rapid analysis in complex environments. As the methods for EV isolation and size measurement appear well accepted among the research community (and have been well reviewed elsewhere), we herein focus on the biological properties of EVs and the specific analytical techniques which can be applied on the nanoscale. In parallel, with the rapid development of labelling technologies and fluorescent dye chemistry, fluorescence is nowadays a versatile tool that offers multiple options for a variety of analytical and imaging modalities. As there is not a standardized workflow for phenotypic studies of EVs, we present an overview of the classic as well as the latest approaches used in the field [19]. In brief, we review analytical strategies involving: nanoparticle tracking analysis, flow cytometry, fluorescence microscopy and super-resolution microscopy, quantitative polymerase chain reaction (PCR), fluorescence lifetime and anisotropy measurements and novel advances in the field of microfluidics and microarrays (Figure 1), and we provide an overview of the labelling technologies used on EVs.

From the techniques listed, fluorescence measurements can be generally divided into two subgroups: A) steady-state and B) time-resolved analysis. A) Steady-state fluorescence spectra result from the excitation of the sample with a continuous light beam and the recording of the emission intensity. In practice, steady-state measurements average the recorded parameters from the initiation until the completion of the phenomenon. B) In time-resolved techniques, data are collected in a time-dependent manner, resulting in a fluorescence decay curve representative of not only the steady-state aspects but also transient information on the phenomenon under study (including quenching, molecular rotation and energy transfer) [20]. Most methods fall in the steady-state category, apart from FLIM and fluorescence anisotropy, in which the lifetime of the fluorophores is used.

Fluorescence provides information about a range of processes, such as the interaction of fluorophores with the solvent or rotational freedom and molecular distances [21]. Here we discuss in detail the potential of fluorescence in the detection and nanometrology of EVs. However, there are also limitations in the use of fluorescence-based techniques for the analysis of biological material that are mainly attributed to interference from an intrinsic fluorescence background signal, the need for labelling, as well as photobleaching and quenching by oxygen. Interestingly, many of these drawbacks are ameliorated by the development of stable fluorophores such as quantum dots (QDs) to minimize photobleaching, as well as the use of far-red dyes to improve tissue penetration and eliminate autofluorescence interference. Indeed, in the case of Stimulated Emission Depletion microscopy (STED) microscopy, photobleaching has been put to good effect in providing the very principle upon which the technique is founded [20,22].

A simple categorization of the physical state of the sample and the associated optical analytical approaches is as follows:

- Analysis of intact EVs, in which the sample can be:
  - in the form of a dynamic suspension (nanoparticle tracking analysis (NTA), flow cytometry, fluorescence anisotropy, live microscopy);
  - captured on a surface (immunomagnetic beads, arrays, microfluidics, microscopy on fixed samples);
- Lysed EVs for molecular analysis (PCR microfluidic systems).

Most techniques analyse either sizing (e.g. Dynamic Light scattering, DLS) or molecular functionality (e.g. flow cytometry, PCR) and there is a need for advanced techniques in the areas of fluorescence NTA, microscopy and microfluidics that offer both size and functional information simultaneously (Figure 1). The potential for EV analysis is not limited by the current techniques, as there are many possible routes for use of complex methods, combining the above, which can be used to answer numerous critical questions about EVs that have not yet been addressed.

**Figure 2.**
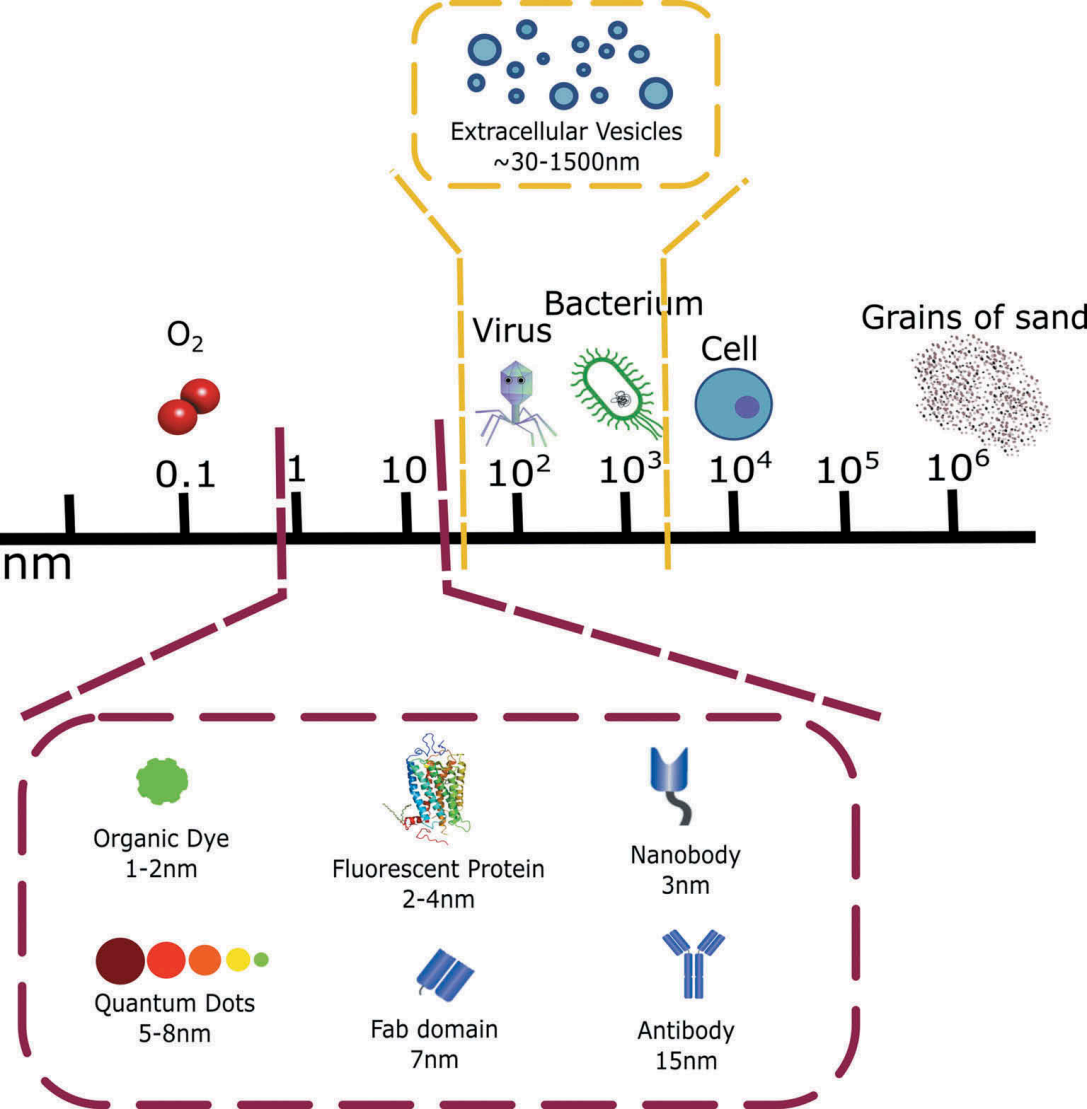
Schematic representation of the relative sizes of EVs and types of fluorescent labels and molecular probes.

### EV labelling technologies

In order to utilize imaging and spectroscopic techniques based on fluorescence, either direct or indirect labels need to be introduced to the EV target sample. Examples include the following options for labelling strategies: (i) staining via the sequestering of free dyes directly into the lipid bilayer via hydrophobic interactions or by genetically encoding fluorescent proteins, (ii) selection of fluorophore for the formation of a covalent chemical bond with a probe which has affinity for a vesicular compartment, and (iii) the selection of a suitable affinity probe such as immunoglobulins, nanobodies or aptamers. The target components can be nucleic acids (RNA; and also DNA, including via apoptotic bodies [10]), membrane lipids and proteins, and intravesicular molecules, some of which are ubiquitous and others showing high specificity for particular EV sub-types. The selection of an appropriate fluorescent reporter depends on factors including selectivity for a tissue compartment, excitation and emission spectra and other physical and biological properties such as stability, photobleaching, possible toxicity to the specimen, intensity of the signal and whether surface-based measurements or EV samples in suspension are being analysed. A brief overview of the various fluorescent probes already described in the literature is shown in Figure 2.

A broad categorization of the main groups of labelling options mentioned earlier is given here in more detail:

***(i) staining via hydrophobic interactions* and genetically encoded fluorescent proteins**

Free fluorescent dyes are commonly used to stain the total EV population non-specifically, either by lipophilic interactions with the membrane or by diffusion and retention inside the vesicles. Some dyes associating with the membrane of human or bacterial EVs used for flow cytometry measurements and fluorescence confocal microscopy for cellular uptake tracking and trafficking include, among others, BODIPY-PC (conjugated with phosphatidylcholine), DiD, Dil, DilC, Octadecyl rhodamine B chloride (R18) [23] dyes from the BODIPY range as well as PKH67 and PKH26 [24–26]. Fluorescent proteins such as enhanced green fluorescent protein (EGFP) can also become endogenous probes for various cell compartments upon appropriate genetic manipulation of cells and importantly, the majority of these proteins and dyes are compatible with live tissue imaging [27]. Some dyes, non-commercialized to date, are wash-free nucleic acid indicators, examples being the membrane permeable dyes acridine orange and thioflavin T [28]. Another recently described dye emitting in the near infrared (IR) can stain EVs in suspension or gives the option to generate fluorescent vesicles from pre-stained cells [29]. An example of a dye that can sense the membrane integrity of EVs is Calcein AM, which becomes fluorescent only after hydrolysis by esterases confined to the inside of non-permeabilised vesicles [26].

For membrane and lipid rafts studies, there is a large variety of fluorophores which intercalate into the lipid bilayer. These are currently used widely on cells and have potential application to vesicles. The most common category is the BODIPY range of dyes which can bind to different parts of the acyl chain of membrane lipids, thus providing images from different depths of the lipid bilayer [30,31]. Environmentally sensitive dyes such as Laurdan (6-lauryl-2-dimethylamino-naphthalene), carbocyanine, DPH and di-4-ANEPPDHQ appear to alter their fluorescence properties according to the polarity of the microenvironment, which corresponds to the relative rigidity of the membrane, or abundance of the lipids in the local domain, thus making it possible to map different membrane regions [32–34].

***(ii) fluorophores used for labelling an affinity probe***

**Red/near-infrared (NIR) fluorescent dyes** offer a number of advantages for EV analysis. In particular, their longer-wavelength excitation usually minimizes both the background endogenous fluorescence from the sample under study and the detection of Rayleigh scattered excitation [35]. Some advantages of red dyes have been brought to bear in medical diagnostics and imaging using indocyanine green (ICG), which fluoresces up to ~900 nm. ICG is approved for intravenous use and widely used to detect cardiac, circulatory, hepatic and ophthalmic conditions [36,37]. Red/NIR aromatic dyes are often based on cyanine, xanthene, oxazine or coumarin groups. Their common limitations are their susceptibility to photobleaching and short fluorescence decay times of ~1 ns, the latter limiting application to binding studies based on fluorescence polarization anisotropy. Recently, a range of more photo-stable red/NIR azadioxatriangulenium (ADOTA) dyes with a fluorescence lifetime of ~20 ns has been reported. The NHS ester of ADOTA has been used with fluorescence anisotropy to study anti-rabbit Immunoglobulin G binding to rabbit Immunoglobulin G; a 150 to 300 kDa increase resulting in a rotational correlation time increase of ~50 to 90 ns [38]. In addition, methyl-ADOTA has been shown to improve precision as compared to ultraviolet dyes by not exciting auto-fluorescence in silica colloids with uniform nanoparticle radii in a range – 3.5 nm to 11 nm – with respect to EVs of similar size, the latter corresponding to a rotation correlation time of ~1400 ns [39].

**Dyes intended for super-resolution microscopy** based on stochastic activation of fluorophores such as Stochastic Optical Reconstruction Microscopy (STORM) are special organic molecules or proteins which have blinking properties (reversible photoswitching) such as the AlexaFluor and the ATTO range of molecules [40]. Techniques involving photoactivation such as PALM usually require fluorescent proteins (e.g. Photoactivatable-GFP) in cases where exogenous cellular expression of the fluorescent protein is possible [41]. More details on the applications of high-resolution microscopy on EVs will be described later.

**Luminescent nanoparticles** such as inorganic QDs absorb light across a specific wavelength region and emit narrower spectral bands of light depending on the composition, size and surface properties of the nanocrystal. Their spectral versatility and capacity for surface modification have made them popular for biomedical applications [42]. Apart from their use as a direct fluorescent agent conjugated with probes of biological relevance, they have also been used as parts of FRET pairs for the detection of nucleic acid sequences as, for instance, they have been coupled with thiazole orange to detect double-stranded oligonucleotides [43]. Pairing quantum dots with an aptamer against the EpCAM molecule and quenchers has been shown to generate a “beacon” that lights up in the presence of the target, thus providing a detection probe for the circulating metastasis indicator, EpCAM [43]. QDs are suitable for NTA studies of EVs, as they show stability in the emitted intensity over long periods of excitation (see also NTA section below). Other bright nanoparticles include nanodiamonds, upconversion nanoparticles and gold nanoparticles, which show optical properties and small size, suitable for EV labelling and single-molecule analysis, such as advanced microscopy and spectroscopy [44].

***(iii) options for affinity-binding reagents***

Although antibodies and Fab domains are being used for specific EV labelling, extrapolating their potential from their well-tried use on cells, the size of EVs suggests that smaller labelling probes can be more effective, as they can potentially increase the label-to-EV ratio. **Nanobodies** are fragments engineered from antibodies found naturally in camelids (camels, llamas) and cartilaginous fish (sharks). They are single-domain components of the variable parts of an antibody, with a weight of 15–25 kDa, significantly smaller than Fab fragments (around 50 kDa) and whole immunoglobulins (around 150 kDa) [45]. Besides their therapeutic applications, nanobodies are also being used to image biological systems [46]. Fusion with fluorescent proteins is a common method to create fluorescent probes (chromobodies) for fixed and live cell imaging with conventional microscopy [47], as well as super-resolution techniques [48]. Conjugation with chemically synthesized chromophores for intraoperative imaging [49] or even with QDs illustrates their applications [50]. With EVs, there are examples of nanobodies designed against CD9, an EV surface marker [51], as well as lipid-nanobody conjugates embedded in the membrane enabling EV targeting [52–54], thus creating possible applications for the immobilization of EVs on arrays, beads or microfluidic systems besides direct fluorescent labelling.

**Nucleic acid aptamers** are synthetic single-stranded DNA or RNA sequences that are selected based on their high specificity for a target species and can be custom-engineered with different labels to amplify the optical signal associated with EV interaction. DNA dendrimers are a type of 3-D macromolecular structures with numerous binding sites, incorporating DNA sequences and have been used as part of an EV capture and detection platform, where the capture of EVs on CD63 magnetic beads triggers the release of a DNA probe which hybridizes on other hairpin structures and initiates a cascade of signal which is finally amplified by the dendrimers [55]. CD63 aptamers have been also conjugated with QDs for the labelling of EVs for super-resolution microscopy [56] and as part of a cancer-derived EV detection system using clinical serum samples [57]. A method for quantifying EVs and selectively labelling specific sub-classes using aptamers has been recently developed with the following principle: the EV population was labelled with a construct coupled to a DNA oligonucleotide sequence and the signal amplified by an isothermal nucleic acid detection assay based on the amplification of DNA fragments and enzymatic generation of fluorescent fragments [58].

## Analytical techniques

### Nanoparticle tracking analysis

Nanoparticle tracking analysis (NTA) is a relatively recently developed (2006) technique, now distributed by Malvern Panalytical Ltd (UK), which can be generally applied to many types of nanoparticles ranging from 30 nm to 1 μm, thus encompassing many EVs [59,60]. In more detail, the sample is a dilute liquid suspension of particles illuminated by a laser beam (Figure 1A). The scattered light from the particles is collected via a microscope objective lens and recorded at video frame-rates, the choice of camera sensitivity depending on whether Rayleigh scattering only or also fluorescence-based tracking is recorded. The software can identify a particle and track its trajectory from one frame to the next, as well as measure its velocity. The value recorded for velocity is applied on the two-dimensional Stokes–Einstein equation to obtain the particle hydrodynamic diameter [61]. NTA measurement is relatively fast (requires a few minutes) with hundreds of individual tracks collated to provide insight into the colloidal composition and provides real-time imaging of particles while the phenotype of the particles can also be determined [60].

This technique is one of the most widely accepted methods for EV size and concentration measurements, and can also be used for EV phenotyping using fluorescent markers. Because of the prolonged exposure of the sample in the laser beam, the most popular fluorescent labelling is via QDs, as they are generally photostable compared to other commercial fluorophores and this strategy is overall considered more sensitive than flow cytometry for particles in the sub-micron size range [62]. A number of research groups have reported the use of NTA for EV phenotyping. Some of the markers studied by the attachment of the relevant antibody to quantum dots are the surface urinary marker CD24 for urine-derived exosomes [63] and CD63 along with EpCAM for EVs isolated from ovarian cancer patients’ blood [64]. EVs from maternal blood were screened for CD63 and placental alkaline phosphatase (PLAP), an indicator of placental exosomes, in order to detect obesity-related gestation complications [65] and in another study, in order to monitor foetal growth restriction [59]. Placental EVs directly isolated from the perfusate of ex vivo tissue were also similarly analysed for the placental marker NDOG2 [62]. An approach different from the QDs system was applied on Jurkat cell-derived EVs which were phenotyped for CD45 by immunocapture with anti-CD45 coated magnetic beads and subsequent measurement of the EV depletion level from the initial population [66].

Regarding the relative advantages of NTA over other techniques discussed later, NTA measurements are relatively fast (require a few minutes), with non-sophisticated sample preparation procedure and provide real-time imaging of unlabelled or labelled particles. It is also particularly sensitive for samples containing small populations of larger particles because the intensity of the scattered light is proportional to the sixth power of the diameter. At the same time, this means that size determination could be less accurate, especially in populations of smaller particles, where dust particles or protein aggregates can interfere in the measurement. This poses an additional point of limitation, related to the sample purity. The presence of large particles reduces the number of the small particles the software can detect [60]. Different size populations can be measured only when there is a 1.5﻿fold difference in their diameter, and because the precision depends on the refractive index of the specimen, it is suggested that samples with high size heterogeneity should be measured more than once [67]. Currently, instruments with two different fluorescence channels are under development, as discussed in the “Future outlooks in EV analysis” section [68].

### Flow cytometry

A versatile technique to characterize labelled or unlabelled cells and particles derived from biologic samples is flow cytometry. The working principle of this technique is the sensing of cells or particles in suspension, streamed through a laser excitation path [69]. Once the cells or particles pass through the excitation source (usually laser beam), the light is scattered in all directions and the information obtained from the orientations of the detected photons can be translated into size (forward scatter) and granularity of the sample (side scatter). The electronic system converts the light signal to a digital readout and a computer gives the operator the options of various plots and histograms to interpret the data which apart from size and granularity, give information on the presence of a fluorescent label on each detected event [70].

Flow cytometry was originally designed for larger particles (typically >200–500 nm), substantially larger than the smaller end of the EV size spectrum, but has been used to count, sort and investigate the phenotype of EVs, although their small size raises a matter of controversy for this technique [71,72]. As it is generally accepted that conventional instruments are optimized for larger particles, there are examples of custom-built flow cytometers or specifically adjusted instrument parameters which are dedicated for EV samples [73–75], giving special emphasis to judicious fluorescent labelling to avoid artefacts resulting from dye aggregation. Some modern instruments can resolve fluorescent particles in the range of 100 nm [76] and it has been shown that imaging and high-resolution flow cytometry offers a new approach in EV analysis [77,78 and 79 respectively].

Another commonly used approach for flow cytometry of EVs is immunocapture on magnetic or non-magnetic beads to form a larger complex which can be detected within the conventional size detection limits of cytometers [80,81]. Some of the most popular EV specific targets are the CD9, CD63 and CD81 molecules on the delimiting EV membrane surface [3]. The immunocomplex can also apply to other techniques within the context of biochemical analysis and microscopy. This can be the basis for further phenotyping studies with the use of special fluorescence-labelled antibodies, but it does not provide direct information on EV size [82–84].

Flow cytometry is a popular method for clinical analyses as it offers the advantages of a rapid and versatile tool for which fluorescent probes are well established and optimized. However, there are concerns regarding the sensitivity of the system as applied to EVs. Alternative techniques such as NTA which are designed for sub-micron particles and offer more sensitive phenotype analysis [62] demonstrate that sectors of the EV population are not detectable by flow cytometry [85], mainly because of the low signal/noise ratio of detection in Rayleigh scattering as smaller particles move through the detection channel. It is also acknowledged that while the more advanced imaging flow cytometers offer a resolution limit of ~100 nm, there are other complementary techniques which cover the vesicle sizes below 100 nm, such as fluorescence Nanoparticle Tracking Analysis, electron microscopy or protein and lipid profiling [67].

### Fluorescence microscopy

With EV diameters ranging from a few nanometres up to a few hundreds of nanometres, the diffraction-limited resolution capabilities of a microscope (usually 200–300 nm) in the study of such small particles are unworkable. However, although the small size of many EVs does not allow their visualization in an unlabelled state by brightfield illumination, the attachment of a fluorophore which emits light enables the imaging of EVs and their interactions with live cells. Imaging by microscopy is particularly useful for the study of the localization of the particles in cells or tissues, or presence of a fluorescently labelled target. The majority of the cases discussed in this section refer to confocal microscopy, in which only a small defined area of the sample is exposed at each time excluding all the out-of-focus light, in contrast to wide-field imaging where the total area of the specimen is illuminated and the precision of the fluorophore localization is lower [86].

Several applications of confocal imaging of EV include the observation of vesicles inside the Golgi apparatus with a size of approximately 50–80 nm [87], and in addition, immobilization of EVs on a solid surface is used to directly visualize the individual vesicles [88], and also their localization in cells after uptake [89]. Cancer cells co-cultured with EVs have been imaged with fluorescence microscopy [90], giving possible implications on uptake mechanisms. An example of live tracking of EVs in a living organism comes from Verweij *et al*. by using zebrafish in which all CD63-positive EVs were fluorescently labelled. This model expressed CD63-pHluorin (emits green fluorescence) and therefore, endogenous EVs were labelled and could be tracked in real time *in vivo* by confocal microscopy [91]. What is more, EVs have been visualized in live tissue by fluorescence optical imaging and multiphoton microscopy in intravital window methods (see Table 1 for a summary of references on live microscopy of EVs).

**Table 1.** Examples of studies on live fluorescence imaging of EVs.

Tissue/Cells imaged	Fluorescent probe on EV	Imaging System	Purpose	Reference
F11 (nervous system) -derived EV on F11 cells	GFP	Confocal microscope	Delivery of neurogenic mRNA	Oh *et al*., 2017^92^
Breast cancer cell-derived EVs on HeLa cells	Gold-carbon quantum dots (GCDs)	TIRF-super resolution microscope	Effect of GCDs on cellular uptake of EVs	Jiang *et al*., 2018^93^
293T (kidney) -derived EVs on Gli36 (glioma) cells and mouse tumours	PalmGFP/tdTomato	Confocal and multiphoton intravital microscope	EV tracking between cells *in vitro* and in tissue	*Lai et al*., 2015
HEK293 (kidney) -derived EVs on HEK293 cells	GFP	Fluorescence microscope	Validation of exosomal surface display system	Stickney *et al*., 2016^94^
Mesenchymal stem cell-derived EVs on renal cells and acute kidney injury mice	DiD and DiI	Small animal fluorescence imaging system	Study of distribution of EVs in kidney injury	Grange *et al*., 2014^95^
Glioblastoma-derived EVs on microglia and monocytes/macrophages in mice	Fluorescent proteins	Multiphoton intravital imaging	Release and uptake of EVs by glioma cells and RNA transfer	van der Vos *et al*., 2016^96^
Mouse lung cancer-derived EVs on lung tumour-bearing mice	DiIC18(5)	Animal fluorescence/bioluminescence imaging	EVs as delivery system for oncolytic virus	Garofalo *et al*., 2018^97^
Breast tumour-derived EV on breast tumour bearing mice	DsRed and GFP	Multiphoton intravital imaging	EV release and uptake in tumour	Zomer *et al*., 2015 98

Among the advantages of fluorescence microscopy are the direct localization of the labelled target within the system of interest (cell, tissue or organism), the ability to track the EVs in real time and the fact that image analysis software can nowadays give accurate quantitative results on colocalization of fluorophores, tracking speed and directionality, etc. In addition, in contrast to other microscopy methods such as STORM, PALM and FRET, there is a wide variety of fluorophores which can be used for confocal imaging. The main limitations of this imaging strategy mainly pertain to the resolution limit, allowing only larger EV to be accurately imaged, and the sample preparation procedure, which can sometimes involve long protocols including fixation. Another issue always appearing in microscopy is the selection of the appropriate target for labelling, and the suitable fluorophore which needs to be stable and resistant to photobleaching from prolonged exposure [86].

### Super-resolution microscopy

An excellent example of the most advanced applications of fluorescence is super-resolution microscopy, where different techniques use distinct properties of fluorescence to break through the light diffraction limit (250 nm axial and 450–700 nm lateral resolution). Among the most popular techniques are Structured Illumination Microscopy (SIM) which is based on the illumination of the specimen by a striped pattern [99] and Stimulated Emission Depletion microscopy (STED), using a second laser to temporarily bleach the fluorophore around the focal point, thus achieving sharp emission from only a small point of the specimen, as described by S. Hell [22]. Controlled or stochastic activation of the fluorophore, “blinking”, introduces another range of methods which push the resolution limit even lower, achieving single-molecule localization (SMLM) [100]. The most common of these are Photoactivation Localization Microscopy (PALM) which employs genetically expressed photoactivatable fluorescent proteins [41] and direct Stochastic Optical Reconstruction Microscopy (dSTORM) where the underlying chemistry causes the fluorophore to blink in a non-controlled pattern reducing the density of the emitting molecules at each time-point. Recording a series of frames in each of which only a few molecules emit, it is possible to precisely localize the fluorescent molecules and by adding all frames together, the total image is reconstructed in super-resolution down to ~20 nm [40,101]. Total Internal Reflection microscopy (TIRF) is a sample illumination method that uses an evanescent wave of light to image the planar surface of the specimen in the areas of contact with the coverslip and is often used in combination with SMLM approaches [102].

EVs have been imaged with PALM and dSTORM to study the interactions of exosomes with neurons in Alzheimer’s disease. CD9-labelled vesicles were found in the cytoplasm and endosomes of dendrites isolated from mouse brain tissue with a precision of 25 nm [103]. In another study, CD63 aptamers were conjugated with QDs to label the EV surface and the EV-QD complex was imaged using the principles of SMLM, showing the potential of QDs as blinking fluorophores [56]. The same group also used TIRF and SMLM to image EV miRNA and membrane receptor both in vesicle preparations and in living cells [104], while miRNA imaging has been reported from Oleksiuk *et al.*, as well [105]. Labelling CD63-targeting antibody with Alexa Fluor 647 and anti-HER2 antibody with Alexa Fluor 488 enabled imaging with TIRF and simultaneous dual colour PALM/dSTORM for breast cancer cell line-derived EVs alone, as well as upon interaction with non-cancer cells [106]. EVs from mesenchymal stem cells were also labelled with PKH26 and with exosome-specific markers and their intracellular localization in bone marrow mesenchymal stem cells was characterized by SIM [26]. STED is also the method chosen in neuroscience for synaptic vesicle imaging, where a synaptic vesicle membrane-specific membrane protein, synaptotagmin was targeted with Atto532-coupled antibodies [107]. A more recent and currently under development method using super-resolution microscopy is intracellular tracking of EV after uptake, as well as sizing (with an approach alternative to NTA, taking advantage of single-molecule fluorescence) [108].

The benefits of super-resolution microscopy and SMLM are by definition the ability to break the resolution limit and image particles non-detectable by conventional microscopy. It is therefore an approach very suitable for EV analysis regarding size, concentration and content, although this does not come without limitations, mainly from the perspective of sample preparation and the variety of imaging modes which are possible. In more depth, although the protocols for fixation and staining of the biological materials are considered well established for confocal microscopy, when it comes to SMLM, the precision of imaging reveals insufficient labelling of all target molecules which can be due to inadequate staining with affinity reagents or inappropriate labelling strategy. In other words, for imaging intended for quantitative analysis, the location of the target epitope on the particle under examination is a point which needs to be carefully considered when choosing the appropriate affinity probe and labelling conditions. Another limitation which was only discovered with the advent of SMLM approaches is the fixation method, as it has been shown that the structure and clustering of the target can vary significantly with different fixation protocols [109]. Moreover, a drawback inherent to STORM and PALM because of the emission mechanism of the fluorophores is that a large number of the conventionally used dyes are not applicable in these methods, narrowing down the availability of fluorescent molecules. Also, specifically referring to STORM, this technique is mostly recommended for fixed samples, as the specimen needs to be embedded in a special buffer with reducing agents – to induce the “blinking” effect of the fluorophores – in which most live cells cannot be maintained healthy for periods of time sufficient for imaging. This poses limitations in EV tracking in live cells and therefore, an alternative approach (such as STED [110]) would be recommended in those cases when the biological system is not compatible with the imaging conditions [111].

### Fluorescence lifetime imaging microscopy

Fluorescence lifetime imaging microscopy (FLIM) differs from steady-state fluorescence microscopy as in the former, the contrast of the image is generated by the difference in the excited state lifetime of the fluorophores rather than the total fluorescence intensity in case of the latter. In addition to fluorescence localization and intensity information provided by steady-state microscopy, FLIM is especially useful for studying the spatial arrangement of biological membranes, as fluorescence lifetime of a dye depends on its microenvironment [112,113].

Imaging membrane microdomains of EVs can be useful in revealing potential mechanisms of interaction with other membranes and especially cells, and has been used by several groups for cellular uptake imaging. EVs from osteosarcoma cells and their interactions with lung tissue have been imaged with FLIM using the dye PKH67, providing high-quality images of the membrane [24]. A detailed study on the uptake mechanisms for different subsets of EVs was presented by Saari and colleagues, in which paclitaxel-loaded exosomes and microvesicles interacting with PC-3 cells were imaged in real time. Paclitaxel was labelled with Oregon Green and the vesicles were generally stained with the membrane dye, DiD. FLIM revealed the localization, concentration and lifetime of the fluorophores within the cells and it was shown that exosomes have a different uptake mechanism from microvesicles [114]. Another application of FLIM is described by Grela *et al*. where the incorporation of the anti-fungal drug amphotericin B in membranes was investigated by treating cell lines with the drug and imaging the secreted EVs, recording both the lifetime and anisotropy. The images reveal the different microdomains on the EV membrane and show that amphotericin replaces cholesterol in the membrane [115].

Overall, as FLIM is based on the lifetime of the fluorophores, it is independent of probe concentration, excitation intensity or photobleaching and can be used even with spectrally similar fluorophores, given that their decay times are different. Moreover, it can directly detect differences in the probe’s microenvironment, without the need for special treatment of the specimen. On the other hand, the main drawbacks are the need for expertise in lifetime spectroscopy in order to understand the data and the special equipment required [116]. Another limitation of FLIM is the slow acquisition times, however, as seen in the “future outlooks in EV analysis” section, there are currently hardware developments which have started addressing the issue, providing faster imaging [117].

### FRET

Förster Resonance Energy Transfer (FRET) is the phenomenon whereby energy from one fluorophore is transferred non-radiatively to another via dipole–dipole interaction when the two molecules show some spectral overlap (resonance) and are practically less than 10 nm apart in space. Thus, these measurements are used to determine molecular distance and interactions between fluorescently labelled targets.

The combination of FRET/FLIM has enabled quantification of the FRET effect using the advantages of time-resolved lifetime spectroscopy and has been useful in the case of HER receptor in breast cancer since the status of dimerization of HER provides more valuable prognostic/diagnostic information than the levels of expression. HER monomers on circulating EVs were immunostained with the FRET pair AlexaFluor 546 and Cy5 and imaged with the above combination of techniques to quantify the different forms of HER [118]. Besides phenotyping studies, FRET pairs of dyes have served as proximity sensors for EV fusion with liposomal membranes by the use of lipids labelled with NBD and rhodamine and subsequent recording of the fluorescence spectra [119]. A combination of fluorescein (FITC) and rhodamine-labelled peptides were loaded on exosomes and FRET was used to measure their proximity on the same vesicles in a study conducted by Gao *et al*., showing potential for loading and targeting vesicles from patients by the attachment of a special peptide on the CD63 surface molecules [120]. An indirect application of FRET on vesicles was the measurement of this effect on cells which contained a fluorescent protein FRET pair for tau protein, upon treatment with exosome-like vesicles. The results showed that the vesicles had an impact on the aggregation of tau, which is implicated in Alzheimer’s disease pathogenesis [121].

Although FRET offers unique imaging information over other microscopy techniques, the principles of Förster resonance bring disadvantages, as the appropriate combination of emitters needs to be carefully selected. It is also observed that the signal-to-noise ratio (SNR) can be low and emission is sensitive to changes of the microenvironment of the fluorescent molecule; however, recent advances in the field are tackling these issues [122].

### qPCR

Quantitative Polymerase Chain Reaction is a technique based on the well-established PCR method, which is enhanced with numeric quantitative data in contrast to the traditional PCR which is semi-quantitative. This method assesses EV populations in total, rather than single EVs, as it requires vesicle lysis. The basis for its inclusion here is the use of fluorescence to detect the amount of nucleic acid produced in every cycle of the reaction. As the majority of EV studies are conducted on mRNA and miRNAs, qPCR is a common method of choice [123].

### Microfluidics and microarrays

#### Microfluidics

Microfluidics-based technologies continue to evolve for biological and analytical applications and there have also been recent innovations in the field of EV detection. Most often, these involve the capture and separation of EVs on a solid substrate surface localized with a microfluidic compartment. There are several strategies for EV immobilization and these can be distinguished as passive (using physicochemical properties and forces such as immunoaffinity, size exclusion and flows) and active (electroactivity, magnetic and acoustic forces). The analytes can be detected by a variety of different methods depending on the desired readout signal. For instance, nucleic acids can be amplified on-chip with PCR-based methods, proteins can be detected with principles similar to enzyme-linked immunosorbent assay (ELISA) assays, or the EVs can be lysed, releasing their contents which can be subsequently processed off-chip for the detection of various components such as Western blots or mass spectrometry [124,125].

EV secretion from single cells has been presented on a microfluidic setup, where the EVs secreted by each cell are captured on CD63 beads and visualized by fluorescence imaging. Antibodies labelled with PE were attached on the EVs captured on microbeads and the values of fluorescence intensity of the beads could indicate the rate of cell secretion, concentration and phenotype of the vesicles [126]. A biochip which promises simultaneous EV extraction from serum and detection of the pancreatic cancer biomarker glypican I mRNA has been reported by Hu *et al*. using a catalysed hairpin DNA circuit (CHDC) on special lipid-polymer nanoparticles. When EVs are captured on the surface of the nanoparticles, EV RNA interacts with the hairpin DNA complex which emits an amplified signal upon hybridization, enabling detection of low levels of mRNA expression [127]. A platform with readout that can be measured on conventional instruments such as a plate reader is also possible with the appropriate spatial arrangement of the CD63-coated surface. Labelled EVs are infused on this surface and the retained EVs are measured for fluorescence intensity, not only for quantitative studies but also for biomarker analysis [128]. A miniaturized alternative version of fluorescence flow cytometry was developed by Friedrich *et al*. where individual EVs were transported in a flow stream and visualized on a microscope. The results on synthetic lipid particles showed potential applications in measurement of concentration and size distribution, as well as dual colour imaging for different markers [129].

Other applications of microfluidic systems have been developed for the capturing and concentration of EVs from biopsies, where the amount of available vesicles is limited (e.g. cerebrospinal fluid). Taking advantage of the natural surface charge of EVs, an ion-selective membrane can be used as a trap for vesicles flowing onto this surface, resulting in maximized concentrations and a surface saturated with EVs for further studies [130]. EV enrichment for mRNA recovery and subsequent analysis has been achieved specifically for tumour-derived EVs using a cocktail of capture antibodies. The device was applied on blood-derived EVs from glioblastoma patients and the RNA analysis confirmed the detection of EGFRvIII mutation, a glioblastoma biomarker [131,132]. The expression and phosphorylation of IGF-1R were measured with immunodetection and chemifluorescence reagents after vesicles from 30 μl of plasma from non-small-cell lung cancer patients were applied on a microfluidic system [133].

The miniaturization of EV capture and detection processes on microfluidic chips has promoted research towards the development of hand-held analysis devices, such as the system reported by Ko *et al*. [134]. The basis of this invention was the coupling of an EV chip on a smartphone camera to detect fluorescence signal enzymatically amplified from an assay similar to ELISA. Detection with a fluorescence ELISA-like assay was the method chosen for plasma EV analysis from cancer patients, after deposition of 2 μl plasma on a special graphene oxide-induced nanoporous material [135]. The results proved that it was possible to discriminate between cancer and healthy patient samples, without the need for processing. In addition, another system used an ELISA-based process to couple EVs with magnetic beads and generate droplets of individual beads, measuring their intensity by fluorescence after enzymatic reaction. This platform has a lower detection limit of 10 vesicles, providing the potential for sensitive EV detection for disease diagnosis [136].

#### Microarrays

The development of antibody arrays on solid surfaces offers high-throughput screening for various EV markers often by detection of fluorescence signal. A cocktail of antibodies against CD9, CD63 and CD81 were used by Jørgensen *et al*. to capture the total EV population from blood plasma on array spots, followed by visualization using fluorescent detection antibodies. This has enabled screening for 21 different EV surface antigens with the use of less than 10 μl of sample [137]. Later on, the same group reported the development of an array with capacity of screening 60 surface antigens from blood plasma without prior processing or enrichment [138]. Another established array, initially developed for leukaemia cells (DotScan), has been successfully implemented on vesicles for phenotypic analysis by fluorescence and chemiluminescence-based immunoassays [139].

### Emerging fluorescence methods with potential application on EVs

In this section, we discuss some non-conventional tools with potential for EV analysis, such as fluorescence anisotropy and other optical methods.

**Fluorescence anisotropy** takes advantage of the polarization properties of fluorescence that can be used to describe rotational diffusion and energy migration. More specifically, when fluorescent molecules are exposed to a polarized excitation source (i.e. light waves vibrating only in a single plane, with all other planes excluded), only the molecules found in the same plane as the polarized light will get excited. Due to Brownian motion and other energy migration phenomena, the excited polarized molecules will fluoresce while rotating back to randomized planes (depolarization) [20]. The depolarization decay curve of the fluorophore can provide information about its rotational pattern, which is highly dependent on the interactions with the local microenvironment. This method is based on fluorescence lifetime instrumentation and the data can be recorded either in steady-state or in a time-resolved manner. The most common time-resolved measurement is the Time-Correlated Single Photon Counting (TCSPC) technique in which very short light pulses (typically picosecond to nanosecond) are used to excite the molecules of the analyte. The distribution in delay times between the excitation pulse and the detection of single fluorescence photons is recorded and a decay curve of the dye is created, illustrating the fluorescence intensity as a function of time [20]. The addition of components which polarize the light (polarizers) on this instrumentation adds the capability of monitoring the rotation of the fluorophore.

The literature shows an extended interest in anisotropic properties of lipid bilayers over several decades, and these approaches have revealed the behaviour of the membranes under different conditions, usually altering the temperature, pH and lipid composition, using liposomes as artificial membrane models [140]. The results prove that depolarization measurements are sensitive to minor changes in the system under different physical and chemical conditions [141–143].

There has been extensive use of this technique in studying lipid rafts of cell membranes even from the 70s [144,145] to the past decade [146,147]. In the field of EVs, researchers have used the technique to examine the behaviour of the vesicle membrane under different physicochemical conditions extracting information about their rigidity. EVs from mast cells and dendritic cells have shown a difference in fluidity with the rise of pH from 5 to 7, suggesting that membrane re-organization takes place from the acidic environment of the multivesicular bodies to the neutral extracellular matrix. In practice, the vesicles were stained with DPH (diphenyl-hexatriene) and their anisotropy was measured in a steady-state system for different pH and temperature points [148]. A similar system using DPH and steady-state depolarization measurement has been used to investigate the membrane microviscosity on epididymosomes, the EVs secreted in the epididymal fluid. The results showed that the proportion of sphingomyelin in the epididymal membrane increases significantly in transit from the first compartments of the epididymis (caput) towards the “tail” (cauda) of the epithelium, and this change in lipid composition is evident by the increase of the membrane rigidity [149], giving implications for use on EVs from other tissues. Another example of steady-state anisotropy measurement for DPH-stained vesicles from ovarian cancer comes from Shenoy *et al*., who studied the membrane order and phase transition of the particles for a range of temperatures and conclude the lamellarity of the vesicles, evident by the broad phase transition points around 37°C [150]. However, there is a lack of publications on EV depolarization measurements using time-resolved anisotropy instrumentation (TCSPC) to date. Time-resolved fluorescence anisotropy can also be applied to FLIM [151].

In this review, we have focused on the phenotypic analysis of EVs highlighting the versatility of fluorescence. It is also important to acknowledge techniques which use principles different from fluorescence, but which are of great significance in EV analysis (e.g. Raman spectroscopy, mass spectroscopy for lipids and proteins analyses, nucleic acid sequencing, small-angle X-ray scattering (SAXS), micronuclear magnetic resonance (microNMR), electron microscopy (EM), cryoEM-tomography and numerous others).

## Future outlooks in EV analysis

There are currently many outstanding questions about the biology of EVs including their routes of biogenesis, release and uptake mechanisms by recipient tissues *in vivo*, as well as their lifetime in the blood circulation. More advanced tools are required to address these properties of EVs and one of the future directions could be the combination of fluorescence with other optical techniques, as well as multiplexed analysis by adding modalities which use different and non-overlapping principles.

For example, in NTA, advances in the method would tackle limitations such as the speed of sample analysis and allow fluorescence screening for multiple surface markers. Currently, instruments with two different fluorescence channels are under development, so that samples labelled with two fluorescent probes can be studied simultaneously, and in addition to this, it is being converted to high-throughput versions, such as with the use of an autosampler [68]. Another recently commercialized platform for EV phenotyping has been developed by Nanoview Biosciences, Boston, and uses single particle interferometric reflectance imaging sensing (SP-IRIS). In more detail, the EVs are captured on an array coated with CD9, CD63 and CD81 and they can then be stained and imaged at the level of single particles. EVs larger than 50 nm can be probed for up to three fluorescent markers simultaneously, while the analysis ensures only EV are measured, in contrast to NTA approaches which count all particles above the detection limit of diameter [152]. An approach similar to this is the single EV analysis (SEA), which is based on the immobilization of EV on a microfluidics compartment followed by staining and imaging of single vesicles. The EV were pre-biotinylated and were captured on a neutravidin surface, on which staining and washing solutions of antibodies could be introduced. The final microscopy imaging was followed by processing of the signal and identification of the fluorescent markers on the single EVs [153].

Also, a recent and promising development for FLIM is the introduction of single-photon avalanche diode (SPAD) array cameras with on-chip pixelated timing capabilities [117,154]. This provides a convenient implementation of multiplexed fluorescence decay measurements [155] as it replaces the need to scan a sample, resulting in fast FLIM measurements in sub-second timescales. This capability presents exciting opportunities for fluorescence lifetime-based live cell imaging and with bespoke nanoprobes for the detection of transient biomarkers such as mRNA [156] in fluid biopsy/flow cytometry.

Looking into the current approaches for EV phenotyping, we conclude that the heterogeneity of size and composition of EVs dictates the use of multiple techniques in order to eliminate the errors resulting from the differences in size. Moreover, the chemistry of fluorescent probes has seen rapid development. There is currently a wide range of options and the selection of the appropriate probe depends on the imaging strategy, as some methods have specific requirements (e.g. STORM, FRET). Because of the growing interest in the use of EVs in clinical analysis, standardization of the phenotyping guidelines is required, as there is significant variability in the techniques in current use. As one of the key questions about extracellular vesicles still remaining is the mechanism of interaction with tissues in vivo, future research could be directed towards the development of live EV imaging and tracking in living models (similar to Verweij *et al*., as mentioned earlier). Finally, the need for clinically applicable devices has started with the development of microfluidics and arrays, which promise rapid, point-of-care analysis of multiple markers simultaneously. Fluorescence-based methodologies for phenotyping EVs in healthcare applications will undoubtedly continue to develop apace and future EV-based diagnostics are highly likely to increase in efficacy and popularity.
